# Egg limitation and individual variation in parasitization risk among hosts in host–parasitoid dynamics

**DOI:** 10.1002/ece3.2916

**Published:** 2017-03-30

**Authors:** Toshinori Okuyama

**Affiliations:** ^1^ Department of EntomologyNational Taiwan UniversityTaipeiTaiwan

**Keywords:** egg limitation, individual variation, individual‐based model, superparasitism

## Abstract

Egg limitation is known to destabilize host–parasitoid dynamics. This study reexamines the effect of egg limitation in light of the individual variation in parasitization risk among hosts (e.g., some hosts are more likely to be parasitized than others). Previous studies have considered egg limitation (predicted as a destabilizing factor) and individual variation among hosts (predicted as a stabilizing factor) in isolation; however, their interaction is not known. An individual‐based model was used to examine the effects of each factor and their interaction. The model‐based analysis shows a clear interaction between egg limitation and individual variation in risk among hosts. Egg limitation can both stabilize and destabilize host–parasitioid dynamics depending on the presence and absence of the risk variation. The result suggests that the population‐dynamic consequences of egg limitation are more complex than previously thought and emphasizes the importance of the simultaneous consideration of multiple ecological factors (with individual‐level details) to uncover potential interactions among them.

## 1 Introduction

Studies on host–parasitoid interactions have offered considerable insight regarding mechanisms that allow consumer‐resource dynamics to persist (Hassell, 2000; Hochberg & Ives, 2000), which is essential information for understanding naturally persisting diverse populations. Density‐dependent self‐limitation (e.g., intraspecific competition) may facilitate stability (Beddington, 1975; Murdoch, Briggs, & Nisbet, 2003), whereas density‐dependent self‐facilitation (e.g., the Allee effect) may destabilize the dynamics. For example, the handling time of consumers induces the latter type of density dependence on the fitness of resources, such as hosts and prey (known as the dilution effect) (Hamilton, 1971), and destabilizes the consumer‐resource dynamics (Hastings, 1998; Murdoch et al., 2003).

Egg limitation (i.e., parasitoids do not possess sufficient eggs to parasitize all encountered hosts) is a destabilizing factor because it induces dilution effects (Getz & Mills, 1996; Shea, Nisbet, Murdoch, & Yoo, 1996). In other words, the per capita survival rate of hosts increases with the host density because parasitoids become increasingly inefficient in parasitizing a given proportion of a host population. In contrast, egg limitation can stabilize host–parasitoid dynamics when it is coupled with density‐dependent variation in the foraging success among parasitoids (Okuyama, 2015; also further discussed in details below). These results are not contradictory and simply indicate that egg limitation and individual variation interact with each other. In fact, even in the presence of density‐dependent individual variation, it is obvious that an extreme level of egg limitation (e.g., each parasitoid can lay only one egg) leads to parasitoid population to extinction due to variaous mortality factors. These results highlight the importance of interactions among ecological factors. Although isolated effects have been well studied for many factors, their potential interactions are hardly understood.

This study examines the importance of egg limitation on population dynamics in the presence of individual variation in the parasitization risk among hosts, which is a different type of individual variation from that discussed earlier. The former is a variation among parasitoids, and the latter is a variation among hosts. When some hosts are more likely to be parasitized (e.g., easier to find), the likelihood of superparasitism increases in those hosts. Superparasitism is common in nature and occurs even when parasitoids have some ability to distinguish parasitized and unparasitized hosts (van Alphen & Visser, 1990; Dorn & Beckage, 2007; Godfray, 1994). Particularly in solitary parasitoids, superparasitism results in egg wastage (but see van Alphen and Visser (1990)). When each parasitoid has a limited number of eggs, a large proportion of eggs might be wasted as a result of enhanced superparasitism. Although the stabilizing effect of individual variation in the parasitization risk among hosts has been well studied (Chesson & Murdoch, 1986; Ives, 1992), its interaction with other ecological factors, such as egg limitation, is unknown. Although egg limitation in parasitoids has been widely studied from behavioral and evolutionary perspectives (Casas et al., 2009; Heimpel & Rosenheim, 1998; Heimpel, Rosenheim, & Mangel, 1996; Rosengeim, 1999; Rosenheim, Heimpel, & Mangel, 2000), we have limited understanding of its population dynamical consequences.

To examine the interaction between egg limitation and individual variation in the parasitization risk among hosts, an individual based model (IBM) was built. Because the focus is on individual variation, individual‐based modeling is an ideal approach. Although there is an existing IBM describing host–parasitoid dynamics (Okuyama, 2015), the model is phenomenological and is limited in its extendibility. In this study, a new mechanistic model was built. First, the previously reported interaction between egg limitation and individual variation in foraging success was reexamined using the new model. Then, the interaction between egg limitation and individual variation in the parasitization risk among hosts was explored. The reexamination of the first type of individual variation helps to strengthen the previous results (e.g., the results are not specific to the particular model structure), and the use of the same model to examine the two different types of individual variation clarifies the interpretation of the egg limitation effect.

## 2 The model

The model considers host–parasitoid interactions in a closed environment. Let *H*_*t*_ and *P*_*t*_ be the densities of hosts and parasitoids, respectively, at time *t*. The dynamics of host–parasitoid interactions are assumed to follow the model presented by Nicholson and Bailey (1935),

(1) $$H_{t+1}\;=\;\lambda H_{t}e^{-aP_{t}}$$

(2) $$P_{t+1}\;=\;H_{t}\left(1\;-\;e^{-aP_{t}}\right)$$

where λ is the host population growth rate (e.g., the contribution of a surviving host to the next generation), and *a* is the attack rate of the parasitoid.

To examine the effect of individual variation, the model (Equations (1) and (2)) was translated into an IBM. In the IBM, each individual behaves according to simple rules (described below), and emergent population level processes are examined by explicitly keeping track of the status of individuals. In each discrete time step (the subscript *t* is omitted in the following descriptions as within‐generation processes are described here), the number of hosts encountered by a parasitoid *u* is simulated from a probability distribution whose mean is *aH* (the specific probability distribution is described below). **p **= (*p*_1_, *p*_2_, …, *p*_*H*_) is the encounter probability vector such that *p*_*i*_ is the probability that a particular encounter is allocated to the *i*th host ($\sum_{i=1}^{H}p_{i}=1$ and 0 ≤ *p*_*i*_ ≤ 1 for all *i*). Each encounter follows the same **p** within the same generation and thus, it is possible that the same host is encountered multiple times by the same parasitoid and/or different parasitoids. The model (Equations (1) and (2)) assumes random encounters such that *p*_*i*_ **= 1/*H* for all *i*, but when there is variation in the parasitization risk among hosts, the elements of **p** are variable and simulated from a probability distribution (described below).

### 2.1 Egg limitation

Each parasitoid can lay a finite number of eggs. In the model, *n*_E_ is the number of eggs laid by a female that survive and reprudce, accounting for mortality factors except for superparasitism. Therefore, *n*_E_ is a fraction of the total number of eggs laid by a female and correlates with egg limitation (i.e., strong egg limitation leads to small *n*_E_). For a parasitoid that encounters *u* hosts, min(*u*,* n*_E_) hosts are randomly picked and parasitized, where min(*u*,* n*_E_) = *u* when *u *< *n*_E_, and min(*u*,* n*_E_) = *n*_E_ when *n*_E_ **< *u*. Thus, when *n*_E_ **< *u*, some hosts are encountered by a parasitoid, but still avoid parasitization as a result of egg limitation. The model (Equations (1) and (2)) assumes that *n*_E_ = ∞, such that all encountered hosts are parasitized. A host that is parasitized one or more times will become a parasitoid in the next generation (i.e., the parasitoid is solitary). If *h*_0_ is the number of hosts that are not parasitized after all the parasitoids had their opportunity to parasitize, then the number of hosts in the next generation is generated from a Poisson distribution with mean *λh*_0_.

### 2.2 Individual variation in foraging success among parasitoids

The number of hosts a parasitoid encounters is a random variable *U* that follows a negative binomial distribution

(3) $$U\;∼\;\mathrm{Negative}\text{‐}\;\;\mathrm{Binomial}\left(\mu ,k\right)$$

such that *E*(*U*) = μ and Var(*U*) = μ+μ^2^/*k*, where *E*(·) and Var(·) describe the expectation and variance, respectively. Therefore, Var(*U*) describes the individual variation in the foraging success among parasitoids. In the model, we set μ = *aH*. The density‐dependent individual variation is introduced by

(4) $$k\;=\;\left(\kappa \;-\;z\right)\;e^{-\beta P}\;+\;z$$

in which β > 0 indicates that, as the parasitoid density increases, Var(*U*) increases. κ describes the degree of individual variation in the absence of a density‐dependent effect. *z* > 0 is set to prevent the variation from becoming unrealistically high. When *k* = ∞, the distribution converges to a Poisson distribution. This notation is used to describe a Poisson distribution, in which κ and β become irrelevant and Var(*U*) is independent of *P*.

Although the principal purpose of this study is not to examine the effects of individual variation in foraging success among parasitoids, the effects of this variation were also examined in the current model to ensure that the same stabilizing effect is produced as that shown in a previous model (Okuyama, 2015). This confirms that the mechanism is robust (i.e., not specific to the particular model considered) and also serves as a test for unexpected behavior of the new model.

### 2.3 Individual variation in parasitization risk among hosts

For a parasitoid with a simulated number of hosts to encounter *u* (a realization of the random variable *U*), the number of encounters for each host is simulated by a multinomial distribution,

(5) Q∼Multinomial(u,p)

where **p** = (*p*_1_, *p*_2_, …, *p*_*H*_) is a probability vector of length *H* defined above. In **Q** = (*Q*_1_, *Q*_2_,…, *Q*_*H*_), *Q*_*i*_ is the number of encounters experienced by the *i*th host such that $\sum_{i=1}^{H}Q_{i}=u$.

When there is variation in the parasitization risk among the hosts, *p*_*i*_ is variable among the hosts. For example, when *p*_1_ > *p*_2_, the first host is more likely to be encountered than the second one. To simulate variation, in each generation, **p** = (*p*_1_, *p*_2_,…, *p*_*H*_) is simulated by a Dirichlet distribution whose parameter is an *H*‐tuple of α (i.e., a symmetric Dirichlet distribution). As α increases, the variation among *p*_*i*_ disappears and converges to *p*_i_ = 1/*H* for all *i*. All parasitoids forage according to the common **p** within the same generation. For convenience, in this study, the situation *p*_1_ = *p*_2_ = … = *p*_*H*_ = 1/*H* (i.e., no individual variation in the parasitization risk among hosts) is represented by α = ∞. α controls individual variation in the parasitization risk among hosts; the smaller the value of α, the greater the variation among hosts.

## 3 Analysis

The stability, considered as persistence (i.e., both the parasitoid and host persist without going to extinction), was quantified. The initial host and parasitoid densities were set to the rounded numbers of λln(λ)/((λ−1)*a*) and ln(λ)/*a*, respectively (i.e., the equilibrium of the Nicholson–Bailey model). The default parameter values used were *a* = 0.001, λ = 1.2, κ = 100, and *z* = 0.1, but the qualitative results described below were not sensitive to the specific combination of these parameters. The parameters regarding the egg limitation *n*_E_, the individual variation in foraging success among parasitoids β, and the individual variation in the parasitization risk among hosts α were varied and their effect on the stability was examined. The model was simulated for 1,000 generations, and the persistence of both species was examined (without the presence of ecological effects such as egg limitation or individual variation, the model does not persist for 100 generations).

## 4 Results

**Figure 1:**
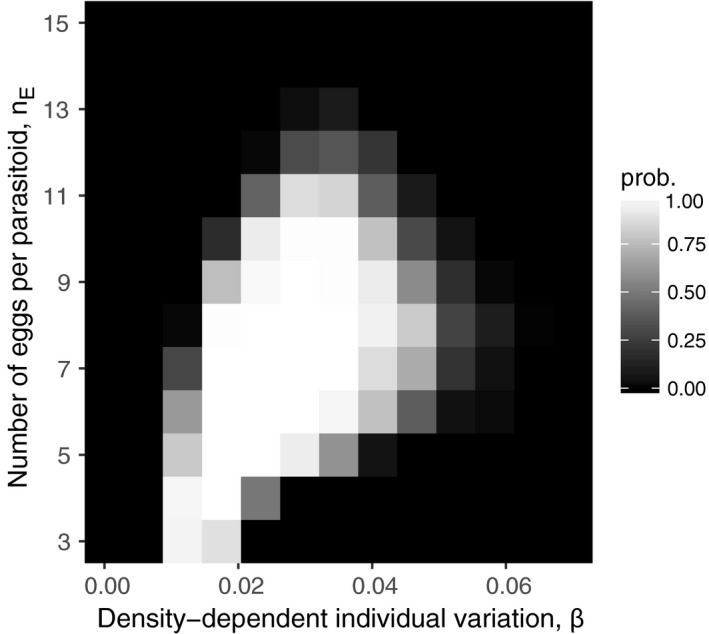
Persistence probability when degree of egg limitation *n*_E_ and strength of density‐dependent variation in the foraging success among parasitoids β are varied. The values were estimated from 100 independent simulation runs

The combination of egg limitation and individual variation in foraging success can stabilize the host–parasitoid dynamics (Figure 1). When there is no egg limitation, persistence is never possible, regardless of the presence of density‐dependent individual variation in the foraging success. Similarly, persistence is never possible when individual variation in the foraging success is density‐independent, regardless of the degree of egg limitation. The two factors interact to stabilize the dynamics (Figure 1).

**Figure 2:**
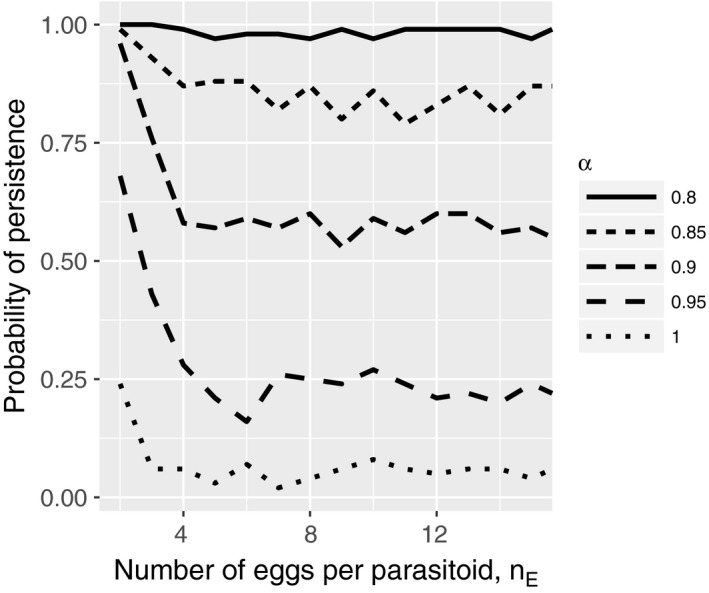
Relation between persistence probability and egg limitation for various levels of variation in the parasitization risk among hosts. The values were estimated from 100 independent simulation runs

**Figure 3:**
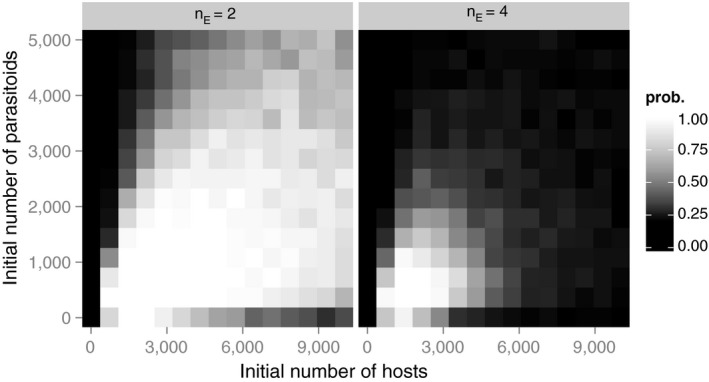
Persistence probability for various combinations of initial host and parasitoid densities. α* *= 0.8, *k *= ∞. The values were estimated from 100 independent simulation runs

In the absence of density‐dependent individual variation in the foraging success (*k* = ∞), variation in the parasitization risk among hosts influences the possibility of persistence, a result that is consistent with previous results (Hassell, 1978; Ives, 1992). When there is no variation in the expected parasitization risk (α* *= ∞), persistence is impossible regardless of the egg limitation level. When α is sufficiently small, persistence is always possible regardless of egg limitation. The effect of egg limitation is demonstrated by two pieces of evidence. First, a weaker variation in the parasitization risk among the hosts (i.e., surrogated by α) is required for persistence when there is a strong egg limitation (Figure 2). Second, even when persistence is possible regardless of the egg limitation (e.g., α* *= 0.8 in Figure 2), the domain of the attractor is larger in the presence of a significant egg limitation (Figure 3).

**Figure 4:**
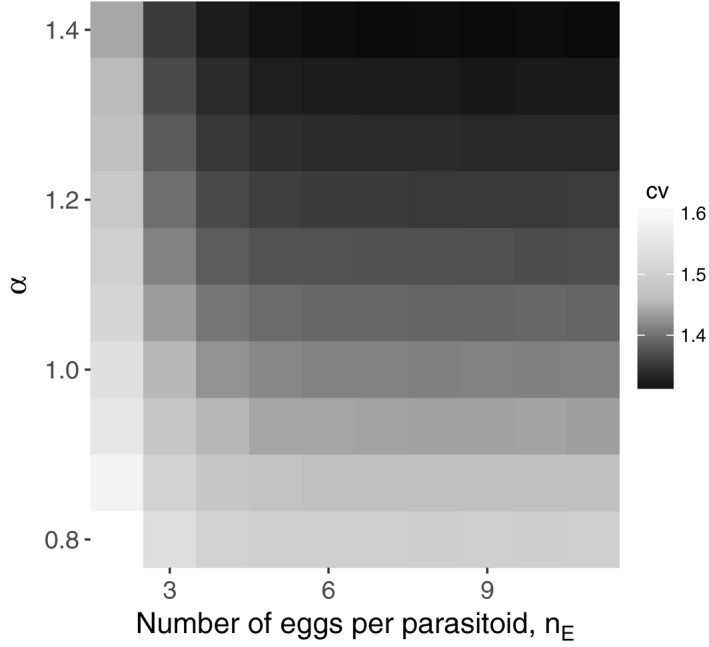
Average coefficient of variation in the number of eggs laid among hosts for various combinations of *n*_E_ and α. *P *= 1,000 and *H *= 2,000. The average was computed from 1,000 simulations

To understand the interaction between egg limitation and individual variation in the parasitization risk, the coefficients of variation in the number of eggs laid among hosts were examined because the coefficient of variation in the parasitization risk is a key index in determining the stability of host–parasitoid dynamics (Chesson & Murdoch, 1986; Ives, 1992). The result shows that egg limitation (e.g., small values of *n*_E_) enhances the coefficient of variation, and the effect is stronger when individual variation in the parasitization risk is high (Figure 4).

## 5 Discussion

Egg limitation has been previously known as a destabilizing factor because it makes parasitoids increasingly more inefficient as the host density increases, a phenomenon known as the dilution effect. Although egg limitation indeed creates a dilution effect, it also has other consequences hidden in conventional mean field models. By IBMs, this study shows the hidden role of egg limitation in host–parasitoid dynamics that emerge primarily from the recognition of individual variation (i.e., individual variation in the foraging success among parasitoids and individual variation in the parasitization risk among hosts).

The mechanism through which egg limitation and density‐dependent individual variation in the foraging success stabilize the dynamics (Figure 1) has been discussed in detail elsewhere (Okuyama, 2013, 2015), and thus it is described only briefly here. In the model, the relationship between the number of hosts encountered and the reproductive success is a concave function because of egg limitation (e.g., a parasitoid cannot lay more than *n*_E_ eggs, no matter how many hosts it encounters). Therefore, for a given expected number of host encounters in the parasitoid population, the realized reproductive output decreases as a result of Jensen's inequality, as variation in the foraging success among parasitoids increases. Consequently, if variation increases with the parasitoid density, it induces a negative density‐dependence on the parasitoid population, stabilizing the dynamics. However, empirical studies that quantify individual‐level foraging success are scarce, and there is little information about how individual variation in the foraging success among consumers changes with the consumer density.

Egg limitation also stabilizes the dynamics when there is individual variation in the parasitization risk among hosts (Figures 2 and 3). For this mechanism to operate, the density‐dependent individual variation in the foraging success discussed above is not needed. When some hosts are more likely to be encountered than others, more eggs are wasted in those hosts that experience a high degree of superparasitism, because only one parasitoid can emerge from a host. Furthermore, when there is egg limitation, it enhances variation in the actual parasitization (i.e., the number of eggs laid) among hosts. In other words, some hosts experience a refuge effect, a stabilizing factor in host–parasitoid dynamics (Hassell, 1978). For example, the oriental fruit fly, *Bactrocera dorsalis* is a host of the pupal parasitoid wasp, *Dirhinus giffardii* (Okuyama, 2016). Because *B. dorsalis* pupate under the ground, *D. giffardii* burrows into the ground and parasitize pupae. Pupae that are near the suface are more likely parasitized (Wu, Huang, Chang, & Chuang, 2014). In this way, pupae that were in deeper soil may experience reduced parasitism risk because of the presence of pupae located near the surface. The variation in pupation depth among hosts may facilitate the persistence of the host–parasitoid dynamics between *B. dorsalis* and *D. giffardii*.

Because the principal focus of this study was to examine the effects of egg limitation in the Nicholson–Bailey model, the IBM did not include sophisticated behavior that is also absent in the Nicholson–Bailey model, such as parasitoids avoiding hosts that are already parasitized (Hubbard, Marris, Reynolds, & Rowe, 1987; Rogers, 1972). If parasitoids avoid superparasitism, variation in the parasitization risk among hosts will decrease. However, superparasitism is readily observed in nature (van Alphen & Visser, 1990; Taylor, 1988; Vinson & Hegazi, 1998; Viser, 1993). The effects described in this study will still operate to some degree as long as there are individual variations among hosts and egg limitation. The mechanistic IBM nonetheless provides a useful starting point for the examination of the effects of other important ecological details.

This study highlights the importance of individual‐level details. Egg limitation is seen to be potentially destabilizing when we focus on its dilution effect. However, egg limitation produces other effects (shown in this study) that can contribute to stability in the presence of individual variation among parasitoids and/or among hosts. Examining each detail of the model empirically might be difficult logistically (e.g., individual‐level foraging sequences), but as surrogate measures, the variation in the number of eggs laid among the hosts (e.g., validation of the pattern shown in Figure 4) can be quantified relatively easily for parasitoids with different *n*_E_ values (e.g., different species) or for some artificially manipulated values of α (e.g., some hosts are placed such that they are easily found). Generating testable predictions in individual variation is an important role of models and will facilitate the connection between theory and data.

## Conflict of interest

None declared.
